# 5,10,15,20-Tetra-2-furylporphyrin

**DOI:** 10.1107/S1600536810014285

**Published:** 2010-04-24

**Authors:** Avijit Ghosh, Ray J. Butcher, Shaikh M. Mobin, M. Ravikanth

**Affiliations:** a Department of Chemistry, Indian Institute of Technology Bombay, Powai, Mumbai 400 076, India; b Department of Chemistry, Howard University, 525 College Street NW, Washington, DC 20059, USA; c National Single Crystal X-ray Diffraction Facility, Indian Institute of Technology Bombay, Powai, Mumbai 400 076, India

## Abstract

Mol­ecules of the title macrocycle, C_36_H_22_N_4_O_4_, are located on an inversion center. The porphyrin ring shows a wave-like conformation with adjacent pyrrole rings tilted above the porphyrin plane and the inter­porphyrin distance is 3.584 (3) Å. The dihedral angles between the *meso*-furyl groups and the porphyrin plane are 38.87 (7) and 48.29 (7)°; these are much smaller than those observed for *meso*-tetra­phenyl­porphyrin, indicating that the *meso*-furyl groups are more inclined towards the porphyrin plane. The decrease in the dihedral angle is due to the presence of intra­molecular hydro­den bonding between the *meso*-fury O atom and the β-pyrrole CH group. Intra­molecular N—H⋯N hydrogen bonds are also present.

## Related literature

The electronic properties of porphyrin macrocycles can be altered by selectively introducing substituents at *meso*- or β-positions, see: Lindsey (2000 ▶). For the effect on the electronic properties of introducing five-membered heterocycles such as thio­phene and furan at the *meso*-position in place of six-membered aryl groups, see: Bhavana & Bhyrappa (2001 ▶); Purushothaman *et al.*, (2001 ▶); Gupta & Ravikanth (2002 ▶, 2003*a* ▶,*b* ▶, 2005 ▶). For the structure of 5,10,15,20-tetra­kis(phen­yl)porphyrin, see: Senge (2000 ▶).
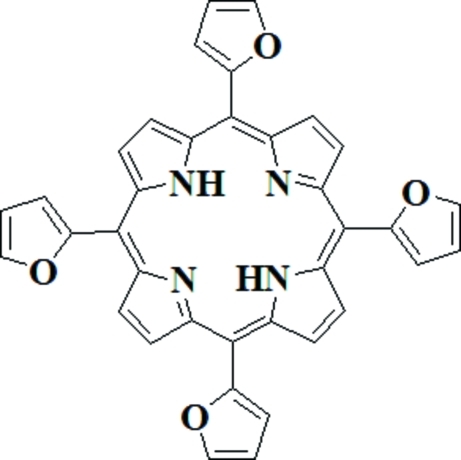


## Experimental

### 

#### Crystal data

C_36_H_22_N_4_O_4_
*M* *_r_* = 574.58
Monoclinic, 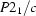

*a* = 9.6068 (4) Å
*b* = 7.3956 (3) Å
*c* = 18.1770 (7) Å
β = 97.419 (4)°
*V* = 1280.63 (9) Å^3^
*Z* = 2
Mo *K*α radiation
μ = 0.10 mm^−1^
*T* = 150 K
0.28 × 0.23 × 0.17 mm

#### Data collection

Oxford Diffraction Xcalibur-S diffractometer
Absorption correction: multi-scan (*CrysAlis RED*; Oxford Diffraction, 2009 ▶) *T*_min_ = 0.973, *T*_max_ = 0.983
14627 measured reflections
4350 independent reflections
2596 reflections with *I* > 2σ(*I*)
*R*_int_ = 0.050

#### Refinement

*R*[*F* ^2^ > 2σ(*F* ^2^)] = 0.057
*wR*(*F* ^2^) = 0.140
*S* = 0.94
4350 reflections
203 parameters
H atoms treated by a mixture of independent and constrained refinement
Δρ_max_ = 0.38 e Å^−3^
Δρ_min_ = −0.30 e Å^−3^

### 

Data collection: *CrysAlis CCD* (Oxford Diffraction, 2009 ▶); cell refinement: *CrysAlis CCD*; data reduction: *CrysAlis RED* (Oxford Diffraction, 2009 ▶); program(s) used to solve structure: *SHELXS97* (Sheldrick, 2008 ▶); program(s) used to refine structure: *SHELXL97* (Sheldrick, 2008 ▶); molecular graphics: *SHELXTL* (Sheldrick, 2008 ▶); software used to prepare material for publication: *SHELXTL*.

## Supplementary Material

Crystal structure: contains datablocks I, global. DOI: 10.1107/S1600536810014285/bt5220sup1.cif

Structure factors: contains datablocks I. DOI: 10.1107/S1600536810014285/bt5220Isup2.hkl

Additional supplementary materials:  crystallographic information; 3D view; checkCIF report

## Figures and Tables

**Table 1:** Hydrogen-bond geometry (Å, °)

*D*—H⋯*A*	*D*—H	H⋯*A*	*D*⋯*A*	*D*—H⋯*A*
N2—H2*N*⋯N1	0.92 (2)	2.29 (2)	2.886 (2)	121.5 (16)
N2—H2*N*⋯N1^i^	0.92 (2)	2.41 (2)	2.9618 (19)	118.1 (15)
C16—H16*A*⋯O2	0.95	2.35	2.855 (2)	113
C17—H17*A*⋯O1^i^	0.95	2.39	2.906 (2)	114

Symmetry code: (i) 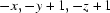.

**Fig. 1.**
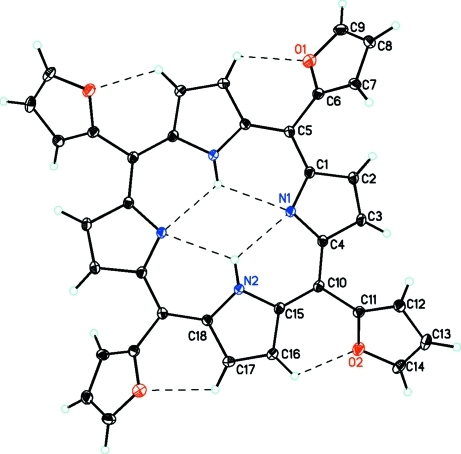
The molecular structure of C36H22N4O4 the showing the atom numbering scheme and 50% probability displacement ellipsoids. The weak C—H···O intramolecular interactions between C—H and O are shown by dashed lines.

**Fig. 2.**
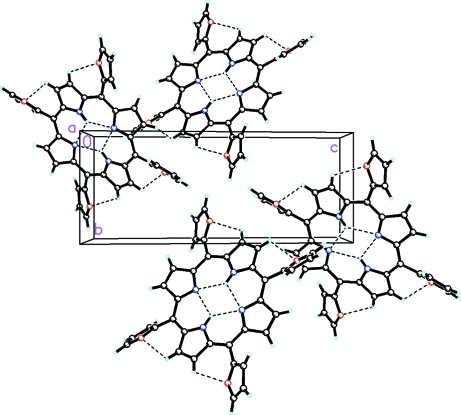
The molecular packing for C36H22N4O4 viewed down the a axis. The weak C—H···O intramolecular interactions between C—H and O are shown by dashed lines.

## supplementary crystallographic information

### Comment

Porphyrin macrocycles are synthetically flexible and by selectively introducing substituents at meso- or β-positions, the electronic properties of the porphyrin ring can be altered at will for any application (Lindsey, 2000). Recently we, and others, have shown that introducing five membered heterocycles such as thiophene and furan at the meso-position in place of six membered aryl groups alter the electronic properties significantly (Bhavana & Bhyrappa, 2001; Purushothaman *et al.*, 2001; Gupta & Ravikanth, 2002, 2003a, 2003b, and 2005). In the crystal structure of the Zn(II) derivative of 5,10,15,20-tetrathienylporphyrin, the structure was shown to correlate with the observed electronic properties (Purushothaman *et al.*, 2001).

In the present paper, we report the crystal structure of 5,10,15,20-tetrakis(2-furyl)porphyrin (I) and compare it with the crystal structure of 5,10,15,20-tetrakis(phenyl)porphyrin (II) (Senge, 2000). The molecular structure of (I) is presented in Fig. 1. The porphyrin plane of (I) displays a wave like conformation with an interplanar porphyrin separation of 3.488 (Å). The aromatic nature of (I) is evident from the observation that the Cα-Cβ distance is greater than the Cβ-Cβ bond distance. The four inner pyrrole N atoms are almost in plane with four meso carbons. The bond distances and bond angles of (I) are altered relative to those of (II) revealing replacing phenyl groups with furyl groups at meso positions changes the porphyrin π-electron delocalization pathway. The dihedral angles of meso-furyl groups with respect to porphyrin plane in (I) are 38.87 (7)° and 48.29 (7)° and those of meso-phenyl groups in (II) are 61.0° and 61.3°. This significant decrease in the dihedral angle in case of (I) is due to presence of intramolecular hydroden bonding between meso-furyl "O" and β-pyrrole "CH". As is clear from Figure 1, the four meso-furyl"O" are involved in hydrogen bonding with two β-pyrrole "CH" which are opposite to each other. This bonding helps in the significant reduction of dihedral angle of meso-furyl groups with the plane of the porphyrin. As a result the meso-furyl groups are inclined more towards the porphyrin plane resulting in extension of π-delocalization of the porphyrin ring to the furyl groups. The observed spectroscopic properties of (I), such as large red shifts in absorption and emission maxima and significant downfield shifts of NH and \b-pyrrole protons in NMR as compared to (II) also in agreement with the enhanced π-delocalization in (I). Thus, the crystal structure presented here indicates that the porphyrin (I) adopts more planar structure as compared to porphyrin (II).

### Experimental

In a 500 ml one necked round bottom flask fitted an with argon bubbler, furan-2-aldehyde (286 mg, 2.98 mmol) and pyrrole (210 ml, 2.98 mmol) in 300 ml of CH_2_Cl_2_ were condensed in the presence of BF_3_.OEt_2_ (120 ml of 2.5 M stock solution) under argon atmosphere for 1 h followed by oxidation with DDQ (674 mg, 2.98 mmol) in open air for additional 45 min. The solvent was removed under reduced pressure and the crude compound was purified by silica gel column chromatography using CH_2_Cl_2_ (62 mg, 12%). M. P. 300°C. Single crystals of (I) suitable for X-ray analysis were obtained by slow evaporation of a dichloromethane/n-hexane solution over a period of one week. Spectroscopic analysis, ^1^HNMR (300 MHz, CDCl3, δ in p.p.m.): -2.59 (s, 2H, NH), 7.04 (m, 4H, furyl), 7.32 (m, 4H, furyl), 8.14 (s, 4H, furyl), 9.16 (s, 8H, β-pyrrole); elemental analysis calculated for C_36_H_22_N_4_O_4_: C,75.25; H, 3.86; N, 9.75%; found: C,75.31; H, 3.92; N, 9.65%: 574.6; found: 574.7(M^+^).

### Refinement

H atoms bonded to C were placed in geometrically idealized positions and constrained to ride on their parent atoms with a C—H distance  of 0.95 Å [U_iso_(H) = 1.2U_eq_(C)]. The H attached to N was refined isotropically.

### Crystal data

**Table:**

C_36_H_22_N_4_O_4_	*F*(000) = 596
*M**_r_* = 574.58	*D*_x_ = 1.490 Mg m^−^^3^
Monoclinic, *P*2_1_/*c*	Mo *K*α radiation, λ = 0.71073 Å
Hall symbol: -P 2ybc	Cell parameters from 1578 reflections
*a* = 9.6068 (4) Å	θ = 2.6–25.1°
*b* = 7.3956 (3) Å	µ = 0.10 mm^−^^1^
*c* = 18.1770 (7) Å	*T* = 150 K
β = 97.419 (4)°	Block, black
*V* = 1280.63 (9) Å^3^	0.28 × 0.23 × 0.17 mm
*Z* = 2	

### Data collection

**Table:**

Oxford Diffraction Xcalibur-S diffractometer	2596 reflections with *I* > 2σ(*I*)
Radiation source: fine-focus sealed tube	*R*_int_ = 0.050
graphite	θ_max_ = 32.7°, θ_min_ = 3.3°
ω scans	*h* = −11→14
Absorption correction: multi-scan (*CrysAlis RED*; Oxford Diffraction, 2009)	*k* = −11→10
*T*_min_ = 0.973, *T*_max_ = 0.983	*l* = −27→27
14627 measured reflections	2 standard reflections every 50 reflections
4350 independent reflections	intensity decay: <2%

### Refinement

**Table:**

Refinement on *F*^2^	Primary atom site location: structure-invariant direct methods
Least-squares matrix: full	Secondary atom site location: difference Fourier map
*R*[*F*^2^ > 2σ(*F*^2^)] = 0.057	Hydrogen site location: inferred from neighbouring sites
*wR*(*F*^2^) = 0.140	H atoms treated by a mixture of independent and constrained refinement
*S* = 0.94	*w* = 1/[σ^2^(*F*_o_^2^) + (0.0704*P*)^2^] where *P* = (*F*_o_^2^ + 2*F*_c_^2^)/3
4350 reflections	(Δ/σ)_max_ < 0.001
203 parameters	Δρ_max_ = 0.38 e Å^−^^3^
0 restraints	Δρ_min_ = −0.30 e Å^−^^3^

### Special details

**Table:**

Geometry. All esds (except the esd in the dihedral angle between two l.s. planes) are estimated using the full covariance matrix. The cell esds are taken into account individually in the estimation of esds in distances, angles and torsion angles; correlations between esds in cell parameters are only used when they are defined by crystal symmetry. An approximate (isotropic) treatment of cell esds is used for estimating esds involving l.s. planes.
Refinement. Refinement of *F*^2^ against ALL reflections. The weighted *R*-factor wR and goodness of fit *S* are based on *F*^2^, conventional *R*-factors *R* are based on *F*, with *F* set to zero for negative *F*^2^. The threshold expression of *F*^2^ > σ(*F*^2^) is used only for calculating *R*-factors(gt) etc. and is not relevant to the choice of reflections for refinement. *R*-factors based on *F*^2^ are statistically about twice as large as those based on *F*, and *R*- factors based on ALL data will be even larger.

### Fractional atomic coordinates and isotropic or equivalent isotropic displacement parameters (Å^2^)

**Table:**

	*x*	*y*	*z*	*U*_iso_*/*U*_eq_	
O1	0.12370 (14)	0.14955 (18)	0.78524 (7)	0.0328 (3)	
O2	0.28868 (13)	1.19097 (16)	0.53303 (7)	0.0277 (3)	
N1	0.14624 (14)	0.57702 (18)	0.58457 (7)	0.0182 (3)	
N2	0.04097 (14)	0.74030 (19)	0.44418 (8)	0.0181 (3)	
H2N	0.018 (2)	0.644 (3)	0.4727 (11)	0.033 (6)*	
C1	0.17490 (17)	0.4903 (2)	0.65124 (9)	0.0190 (3)	
C2	0.25816 (18)	0.6045 (2)	0.70508 (9)	0.0228 (4)	
H2A	0.2896	0.5754	0.7554	0.027*	
C3	0.28217 (18)	0.7587 (2)	0.67017 (9)	0.0223 (4)	
H3A	0.3328	0.8608	0.6910	0.027*	
C4	0.21545 (17)	0.7390 (2)	0.59420 (9)	0.0182 (3)	
C5	0.12791 (17)	0.3159 (2)	0.66826 (9)	0.0183 (3)	
C6	0.19861 (19)	0.2322 (2)	0.73637 (9)	0.0211 (3)	
C7	0.33718 (18)	0.2124 (2)	0.75868 (9)	0.0232 (4)	
H7A	0.4116	0.2566	0.7340	0.028*	
C8	0.3506 (2)	0.1135 (3)	0.82580 (11)	0.0320 (4)	
H8A	0.4358	0.0800	0.8551	0.038*	
C9	0.2214 (2)	0.0765 (3)	0.84007 (11)	0.0334 (5)	
H9A	0.1992	0.0098	0.8817	0.040*	
C10	0.22148 (16)	0.8718 (2)	0.53867 (9)	0.0184 (3)	
C11	0.32446 (18)	1.0178 (2)	0.55473 (9)	0.0199 (3)	
C12	0.45911 (18)	1.0137 (2)	0.58713 (9)	0.0227 (4)	
H12A	0.5096	0.9103	0.6068	0.027*	
C13	0.51107 (19)	1.1943 (3)	0.58618 (10)	0.0287 (4)	
H13A	0.6025	1.2347	0.6053	0.034*	
C14	0.4052 (2)	1.2961 (2)	0.55300 (10)	0.0284 (4)	
H14A	0.4103	1.4226	0.5446	0.034*	
C15	0.13864 (16)	0.8702 (2)	0.46911 (9)	0.0185 (3)	
C16	0.13469 (18)	1.0021 (2)	0.41186 (9)	0.0231 (4)	
H16A	0.1932	1.1058	0.4125	0.028*	
C17	0.03278 (18)	0.9546 (2)	0.35596 (10)	0.0226 (4)	
H17A	0.0057	1.0214	0.3118	0.027*	
C18	−0.02633 (17)	0.7862 (2)	0.37546 (9)	0.0183 (3)	

### Atomic displacement parameters (Å^2^)

**Table:**

	*U*^11^	*U*^22^	*U*^33^	*U*^12^	*U*^13^	*U*^23^
O1	0.0354 (8)	0.0311 (7)	0.0315 (7)	−0.0065 (6)	0.0025 (6)	0.0025 (6)
O2	0.0274 (7)	0.0201 (6)	0.0345 (7)	−0.0029 (5)	0.0002 (5)	−0.0029 (5)
N1	0.0185 (7)	0.0161 (6)	0.0193 (7)	−0.0016 (5)	−0.0006 (5)	−0.0015 (5)
N2	0.0181 (7)	0.0163 (7)	0.0193 (7)	−0.0023 (6)	0.0005 (5)	−0.0004 (5)
C1	0.0193 (8)	0.0179 (8)	0.0195 (8)	−0.0011 (7)	0.0010 (6)	−0.0024 (6)
C2	0.0244 (9)	0.0252 (9)	0.0178 (8)	−0.0035 (7)	−0.0015 (6)	−0.0026 (7)
C3	0.0222 (9)	0.0231 (8)	0.0204 (8)	−0.0048 (7)	−0.0015 (6)	−0.0040 (7)
C4	0.0158 (8)	0.0181 (8)	0.0204 (8)	0.0007 (6)	0.0010 (6)	−0.0024 (6)
C5	0.0171 (8)	0.0191 (8)	0.0183 (8)	0.0003 (6)	0.0011 (6)	−0.0013 (6)
C6	0.0269 (9)	0.0165 (8)	0.0194 (8)	−0.0013 (7)	0.0013 (6)	−0.0023 (6)
C7	0.0192 (8)	0.0235 (9)	0.0264 (9)	0.0014 (7)	0.0008 (7)	0.0066 (7)
C8	0.0348 (11)	0.0229 (9)	0.0337 (10)	−0.0003 (8)	−0.0126 (8)	0.0036 (8)
C9	0.0527 (14)	0.0231 (9)	0.0229 (9)	−0.0074 (9)	−0.0005 (9)	0.0051 (8)
C10	0.0163 (8)	0.0162 (7)	0.0226 (8)	0.0003 (6)	0.0024 (6)	−0.0024 (6)
C11	0.0230 (9)	0.0166 (8)	0.0205 (8)	−0.0017 (7)	0.0041 (6)	−0.0019 (6)
C12	0.0200 (9)	0.0233 (8)	0.0243 (8)	0.0014 (7)	0.0010 (7)	0.0008 (7)
C13	0.0206 (9)	0.0346 (10)	0.0311 (10)	−0.0094 (8)	0.0041 (7)	−0.0100 (8)
C14	0.0331 (11)	0.0193 (9)	0.0340 (10)	−0.0099 (8)	0.0087 (8)	−0.0073 (8)
C15	0.0143 (8)	0.0178 (8)	0.0234 (8)	−0.0011 (6)	0.0024 (6)	−0.0013 (6)
C16	0.0230 (9)	0.0198 (8)	0.0258 (9)	−0.0051 (7)	0.0002 (7)	0.0022 (7)
C17	0.0243 (9)	0.0202 (8)	0.0228 (8)	−0.0021 (7)	0.0010 (7)	0.0040 (7)
C18	0.0186 (8)	0.0178 (8)	0.0181 (8)	0.0005 (6)	0.0008 (6)	−0.0001 (6)

### Geometric parameters (Å, °)

**Table:**

O1—C6	1.359 (2)	C7—C8	1.414 (2)
O1—C9	1.387 (2)	C7—H7A	0.9500
O2—C11	1.371 (2)	C8—C9	1.329 (3)
O2—C14	1.373 (2)	C8—H8A	0.9500
N1—C1	1.367 (2)	C9—H9A	0.9500
N1—C4	1.371 (2)	C10—C15	1.404 (2)
N2—C18	1.373 (2)	C10—C11	1.468 (2)
N2—C15	1.378 (2)	C11—C12	1.351 (2)
N2—H2N	0.92 (2)	C12—C13	1.427 (3)
C1—C5	1.414 (2)	C12—H12A	0.9500
C1—C2	1.452 (2)	C13—C14	1.344 (3)
C2—C3	1.340 (2)	C13—H13A	0.9500
C2—H2A	0.9500	C14—H14A	0.9500
C3—C4	1.453 (2)	C15—C16	1.423 (2)
C3—H3A	0.9500	C16—C17	1.363 (2)
C4—C10	1.415 (2)	C16—H16A	0.9500
C5—C18^i^	1.398 (2)	C17—C18	1.432 (2)
C5—C6	1.470 (2)	C17—H17A	0.9500
C6—C7	1.349 (2)	C18—C5^i^	1.398 (2)
			
C6—O1—C9	106.18 (15)	C8—C9—O1	109.99 (17)
C11—O2—C14	106.71 (14)	C8—C9—H9A	125.0
C1—N1—C4	104.95 (13)	O1—C9—H9A	125.0
C18—N2—C15	110.34 (14)	C15—C10—C4	124.46 (15)
C18—N2—H2N	125.5 (13)	C15—C10—C11	118.31 (15)
C15—N2—H2N	123.7 (13)	C4—C10—C11	117.21 (14)
N1—C1—C5	126.03 (15)	C12—C11—O2	109.69 (15)
N1—C1—C2	110.81 (14)	C12—C11—C10	130.76 (16)
C5—C1—C2	123.13 (15)	O2—C11—C10	119.51 (14)
C3—C2—C1	106.82 (15)	C11—C12—C13	106.88 (16)
C3—C2—H2A	126.6	C11—C12—H12A	126.6
C1—C2—H2A	126.6	C13—C12—H12A	126.6
C2—C3—C4	106.44 (15)	C14—C13—C12	106.52 (16)
C2—C3—H3A	126.8	C14—C13—H13A	126.7
C4—C3—H3A	126.8	C12—C13—H13A	126.7
N1—C4—C10	125.42 (14)	C13—C14—O2	110.19 (16)
N1—C4—C3	110.87 (14)	C13—C14—H14A	124.9
C10—C4—C3	123.71 (15)	O2—C14—H14A	124.9
C18^i^—C5—C1	125.99 (15)	N2—C15—C10	125.79 (15)
C18^i^—C5—C6	117.66 (14)	N2—C15—C16	106.53 (14)
C1—C5—C6	116.27 (14)	C10—C15—C16	127.66 (15)
C7—C6—O1	109.88 (15)	C17—C16—C15	108.49 (15)
C7—C6—C5	129.05 (16)	C17—C16—H16A	125.8
O1—C6—C5	120.94 (15)	C15—C16—H16A	125.8
C6—C7—C8	107.00 (16)	C16—C17—C18	108.02 (15)
C6—C7—H7A	126.5	C16—C17—H17A	126.0
C8—C7—H7A	126.5	C18—C17—H17A	126.0
C9—C8—C7	106.94 (16)	N2—C18—C5^i^	126.57 (15)
C9—C8—H8A	126.5	N2—C18—C17	106.56 (14)
C7—C8—H8A	126.5	C5^i^—C18—C17	126.81 (15)
			
C4—N1—C1—C5	−178.98 (16)	N1—C4—C10—C11	−167.21 (15)
C4—N1—C1—C2	2.96 (18)	C3—C4—C10—C11	13.4 (2)
N1—C1—C2—C3	−1.3 (2)	C14—O2—C11—C12	−0.13 (18)
C5—C1—C2—C3	−179.43 (16)	C14—O2—C11—C10	−178.14 (14)
C1—C2—C3—C4	−0.86 (19)	C15—C10—C11—C12	−136.32 (19)
C1—N1—C4—C10	177.00 (15)	C4—C10—C11—C12	42.0 (2)
C1—N1—C4—C3	−3.52 (18)	C15—C10—C11—O2	41.2 (2)
C2—C3—C4—N1	2.8 (2)	C4—C10—C11—O2	−140.43 (15)
C2—C3—C4—C10	−177.70 (15)	O2—C11—C12—C13	0.29 (19)
N1—C1—C5—C18^i^	−11.1 (3)	C10—C11—C12—C13	177.99 (16)
C2—C1—C5—C18^i^	166.76 (16)	C11—C12—C13—C14	−0.3 (2)
N1—C1—C5—C6	165.50 (15)	C12—C13—C14—O2	0.3 (2)
C2—C1—C5—C6	−16.7 (2)	C11—O2—C14—C13	−0.09 (19)
C9—O1—C6—C7	0.23 (19)	C18—N2—C15—C10	176.59 (15)
C9—O1—C6—C5	176.41 (15)	C18—N2—C15—C16	−1.62 (18)
C18^i^—C5—C6—C7	126.53 (19)	C4—C10—C15—N2	−0.6 (3)
C1—C5—C6—C7	−50.3 (2)	C11—C10—C15—N2	177.63 (15)
C18^i^—C5—C6—O1	−48.8 (2)	C4—C10—C15—C16	177.22 (16)
C1—C5—C6—O1	134.29 (16)	C11—C10—C15—C16	−4.5 (3)
O1—C6—C7—C8	−0.7 (2)	N2—C15—C16—C17	2.51 (19)
C5—C6—C7—C8	−176.46 (17)	C10—C15—C16—C17	−175.66 (17)
C6—C7—C8—C9	0.9 (2)	C15—C16—C17—C18	−2.4 (2)
C7—C8—C9—O1	−0.8 (2)	C15—N2—C18—C5^i^	177.44 (16)
C6—O1—C9—C8	0.4 (2)	C15—N2—C18—C17	0.17 (18)
N1—C4—C10—C15	11.0 (3)	C16—C17—C18—N2	1.42 (19)
C3—C4—C10—C15	−168.37 (16)	C16—C17—C18—C5^i^	−175.84 (16)

Symmetry codes: (i) −*x*, −*y*+1, −*z*+1.

### Hydrogen-bond geometry (Å, °)

**Table:**

*D*—H···*A*	*D*—H	H···*A*	*D*···*A*	*D*—H···*A*
N2—H2N···N1	0.92 (2)	2.29 (2)	2.886 (2)	121.5 (16)
N2—H2N···N1^i^	0.92 (2)	2.41 (2)	2.9618 (19)	118.1 (15)
C16—H16A···O2	0.95	2.35	2.855 (2)	113
C17—H17A···O1^i^	0.95	2.39	2.906 (2)	114

Symmetry codes: (i) −*x*, −*y*+1, −*z*+1.
